# Mitochondria transfer for myelin repair

**DOI:** 10.1177/0271678X251325805

**Published:** 2025-03-13

**Authors:** Sabah Mozafari, Luca Peruzzotti-Jametti, Stefano Pluchino

**Affiliations:** 1 Department of Clinical Neurosciences and National Institute for Health Research (NIHR) Biomedical Research Centre, University of Cambridge, Cambridge, UK; 2 Department of Metabolism, Digestion and Reproduction, Imperial College London, London, UK

**Keywords:** Extracellular vesicles (EVs), cell-free biotherapy, demyelination, mitochondria transfer, neuroinflammation

## Abstract

Demyelination is a common feature of neuroinflammatory and degenerative diseases of the central nervous system (CNS), such as multiple sclerosis (MS). It is often linked to disruptions in intercellular communication, bioenergetics and metabolic balance accompanied by mitochondrial dysfunction in cells such as oligodendrocytes, neurons, astrocytes, and microglia. Although current MS treatments focus on immunomodulation, they fail to stop or reverse demyelination’s progression. Recent advancements highlight intercellular mitochondrial exchange as a promising therapeutic target, with potential to restore metabolic homeostasis, enhance immunomodulation, and promote myelin repair. With this review we will provide insights into the CNS intercellular metabolic decoupling, focusing on the role of mitochondrial dysfunction in neuroinflammatory demyelinating conditions. We will then discuss emerging cell-free biotherapies exploring the therapeutic potential of transferring mitochondria via biogenic carriers like extracellular vesicles (EVs) or synthetic liposomes, aimed at enhancing mitochondrial function and metabolic support for CNS and myelin repair. Lastly, we address the key challenges for the clinical application of these strategies and discuss future directions to optimize mitochondrial biotherapies. The advancements in this field hold promise for restoring metabolic homeostasis, and enhancing myelin repair, potentially transforming the therapeutic landscape for neuroinflammatory and demyelinating diseases.

## Introduction

The central nervous system (CNS) is a highly dynamic and interconnected cellular network with an inherent capacity to maintain its metabolic homeostasis under physiological condition, and to a lesser extent under distress.^1^ The efficiency of CNS energy metabolism is facilitated by the rapid conduction of nerve impulses through the lipid-rich myelin structure produced by oligodendroglia, which rely on mitochondrial function to produce myelin sheaths.^2^ Mitochondrial dysfunction in various neural and immune cells has been linked to neuroinflammation, impaired intercellular metabolic coupling, and axonal loss in demyelinating diseases such as multiple sclerosis (MS).^3^ Moreover, mitochondria–lysosome crosstalk, mediated by AMPA (α-amino-3-hydroxy-5-methyl-4-isoxazolepropionic acid) and mTORC1 (mammalian target of rapamycin complex 1) pathways, is disrupted in several inflammatory neurodegenerative diseases associated with metabolic imbalance such as Parkinson and Alzheimer diseases.^4^ These data underscore the critical role of mitochondrial function and intercellular signaling pathways in maintaining CNS integrity, highlighting potential therapeutic targets for mitigating neurodegenerative and demyelinating diseases.

Mitochondria, referred to as “the processor of the cell”,^5^ play multifaceted metabolic and hemostatic roles in oligodendrocytes, neurons, microglia and astrocytes.

**Figure 1.**
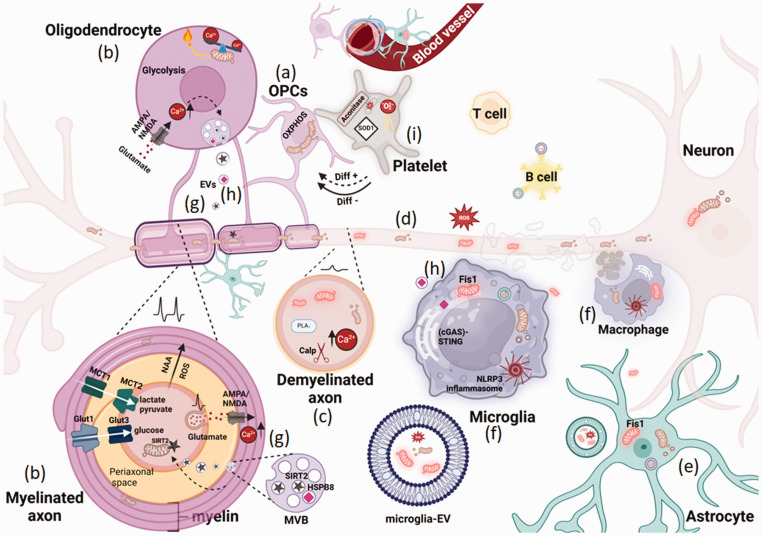
Bioenergetic failure and mitochondria impairment in inflammatory demyelinating lesions. Oligodendrocyte precursor cells (OPCs) contain elongated mitochondria with active oxidative phosphorylation (OXPHOS), which is crucial for myelin generation (a). Oligodendrocytes provide metabolic and nutritional support to neurons by transferring lactate, pyruvate, and glucose to axons. Mitochondria in oligodendrocytes and myelin support fatty acid synthesis, β-oxidation, and recycling of neuron-derived N-acetylaspartate (NAA) and glutamate, while also regulating neuron-derived reactive oxygen species (ROS) activity and maintaining oligodendrocyte calcium homeostasis (b). In demyelinating lesions, oligodendrocyte metabolic, nutritional, and structural support is lost and bioenergetic disruption in axons results from energy failure due to reduced ATP synthesis in axonal mitochondria, caused by impaired lactate and glucose export from oligodendrocytes and astrocytes, and axonal calcium imbalance, which leads to structural axonal damage through activation of calpains, phospholipases, and other enzymes (c). Myelin debris containing inhibitory materials exacerbates neuroinflammation at the lesion site. In response, neurons transport more mitochondria (d) toward demyelinated axons (axonal response to mitochondrial demyelination, or ARMD). Mitochondrial impairment in microglia and macrophages is linked to inflammatory pathways, including the NLRP3 inflammasome and cGAS-STING, which leads to the formation and release of Fis1-mediated fragmented mitochondria into the neural environment, activating A1 astrocytes and contributing to the propagation of inflammation and respiratory stress in lesions (e–f). The release of glutamate following neuronal activity stimulates AMPA/NMDA receptors in oligodendrocytes, transiently increasing oligodendrocyte calcium levels, which can trigger the release of supporting extracellular vesicles (EVs) containing metabolites and enzymes like SIRT2 (g) from multivesicular bodies (MVBs). Neurons take up these vesicles, enhancing their metabolic and mitochondrial function. Furthermore, oligodendrocyte-derived EVs containing HSPB8 can have an immunomodulatory effect on microglia (g–h). Additionally, platelets with impaired mitochondrial function are found in close contact with OPCs in MS lesions (i) and while low levels of circulating platelets stimulate OPC differentiation, higher levels in progressive demyelination have an inhibitory effect (i). Created with BioRender.com.

In oligodendrocytes, mitochondria support energy production and metabolic recycling.^6^ Immature oligodendrocytes rely on dense, elongated mitochondria for oxidative phosphorylation (OXPHOS) to meet the high energy demands of development (Figure 1(a)), while mature oligodendrocytes primarily use glycolysis, featuring smaller, fragmented mitochondria with reduced OXPHOS activity.^2,7^ Mitochondria promote fatty acid synthesis and β-oxidation to support myelin generation and adaptation, recycle neuron-derived N-acetylaspartate (NAA) and glutamate to sustain amino acid metabolism, and buffer calcium to facilitate myelin plasticity at paranodal loops during dynamic changes^2^ (Figure 1(b)). Mitochondrial DNA (mtDNA) damage in oligodendrocytes has been implicated in chronic demyelination and axonal degeneration,^8^ emphasizing mitochondria’s pivotal role in oligodendrocyte development, myelin maintenance, and axo-glial metabolic adaptation.

Neuronal mitochondria are essential for maintaining axonal integrity and meeting the energy demands during myelination and remyelination.^9^ The main mechanisms underlying axonal bioenergetic disruption in demyelinating diseases include: (i) energy failure stemming from the inability of oligodendrocytes to generate and export lactate and pyruvate to axons, leading to reduced adenosine triphosphate (ATP) synthesis in axonal mitochondria; (ii) subsequent failure of ion transporters to maintain proper Na^+^ and K^+^ gradients, impairing action potential propagation; and (iii) axonal Ca^2+^ imbalance, which triggers overactivation of calpains, phospholipases, and other enzymes that ultimately result in structural damage to axons^10^ (Figure 1(c)). In demyelinated lesions, a compensatory increase in axonal mitochondrial mobilization, referred to as the axonal response of mitochondria to demyelination (ARMD), acts as a neuroprotective mechanism^11^ (Figure 1(d)). However, its effectiveness is reduced under inflammatory conditions, such as those observed in experimental autoimmune encephalomyelitis (EAE), an animal model of MS.^12^ Proteomic studies revealed distinct mitochondrial responses to demyelination in white versus gray matter lesions, with white matter exhibiting upregulation of mitochondrial proteins linked to adaptive response and gray matter showing downregulation associated with neuronal loss.^13^

Moreover, disruption of fatty acid oxidation in astrocyte mitochondria has been implicated in the initiation of neuroinflammation, driving neurodegenerative processes.^14^ Comparing data from iPSC-derived astrocytes of three individuals with MS and three unaffected controls revealed several differences in MS astrocytes, including enrichment of neurodegeneration-associated genes, increased mitochondrial fission (Figure 1(e)), elevated oxidative stress, impaired glutamate handling, and altered metabolism with deficiencies in amino acid catabolism and increased sphingolipid metabolism.^15^

Additionally, expression of neurotoxic proteins in microglia alone was sufficient to release fragmented, dysfunctional mitochondria into the neuronal environment, triggering neuronal death and activating astrocytes to the neurotoxic A1 state, which propagate injury.^16^ The balance between damaged and functional mitochondria released by microglia, and the resulting neuronal injury, is regulated by mitochondrial protein fission 1 (Fis1)-mediated mitochondrial fragmentation in glial cells.^16^ Furthermore, activation of inflammatory pathways, such as the NLRP3 (nucleotide-binding domain leucine-rich repeat family pyrin domain containing 3) inflammasome in monocytes and microglia,^17,18^ and the cyclic guanosine monophosphate–adenosine monophosphate (GMP-AMP) synthase cyclic GMP‐AMP synthase (cGAS)–stimulator of interferon genes (STING) pathway,^19^ is linked to increased mitochondrial damage and respiratory stress in MS (Figure 1(f)). Interestingly, inhibition of mitochondria complex I in microglia ameliorates the clinical scores of the EAE animal model^20^ highlighting the key regulatory potential of microglia mitochondria in alleviation of inflammatory demyelinating conditions. Altogether, these studies demonstrate how disruptions in various axo-glial energy-saving and metabolic compensatory mechanisms, coupled with mitochondrial dysfunction, lead to prolonged bioenergetic deficits in axons and glia, ultimately driving irreversible axonal damage and the progression of MS.

Intercellular mitochondrial exchange represents a unique mode of cell-to-cell communication enabling the transfer of functional or damaged organelles between cells through various mechanisms. Key pathways include tunneling nanotubes (TNTs), extrusion, cytoplasmic fusion, connexin-mediated gap junctions, and extracellular vesicles (EV)-mediated transfer.^21^ A recent study demonstrated that deleting the heparan sulfate (HS) biosynthetic gene exostosin glycosyltransferase (*Ext*)1 in myeloid cells reduces mitochondrial uptake by white adipose tissue macrophages, leading to increased obesity and disrupted metabolic homeostasis due to impaired immune-metabolic crosstalk.^22^ This study shows that intercellular mitochondrial transfer, involving several systems, mediates metabolic balance. Changes in mitochondrial dynamics within a cell type require necessary adjustments in its cellular microenvironment to address bioenergetic demands and maintain homeostasis. Whatever the mechanism or cellular origin, damaged mitochondria and impaired intercellular mitochondrial transfer among different neural cell types are linked to reduced energy supply, metabolic dysfunction and myelin pathology, in neuroinflammatory, demyelinating contexts leading to axonal loss.^23^

Cell-to-cell exchange of mitochondria has gained significant attention for its role in maintaining energy metabolism, metabolic coupling, and supporting immune and axoglial functions^24,25^—processes that are particularly perturbed in primary and secondary neurodegenerative demyelination.^26^ In this complex pathological environment, two primary mitochondria-based strategies have been identified through which various key players strive to manage the escalating crisis: (i) the exchange of healthy mitochondria with neighboring cells, aiming to restore balance and prevent suffering from the burden of widespread mitochondrial deficiency; and (ii) the unavoidable removal of dysfunctional mitochondria as a survival mechanism in the pursuit of metabolic balance. While both of these mechanisms may occur simultaneously, the net outcome is determined by the overall function of all the system players involved, in balancing mitochondrial dynamics.

Specific mitochondrial components or regulators have been targeted with small molecules or compounds in different neuroinflammatory conditions, including MS.^27^ However, emerging biotherapies that utilize biological materials, such as stem cells or their cell-free products, including EVs or mitochondria, have recently shown promise for treating diseases associated with mitochondrial dysfunction. Targeting mitochondrial intercellular communication through EV-based or free mitochondria approaches has gained significant attention for its potential to offer new therapeutic strategies for CNS diseases with metabolic dysfunction. While mitochondrial delivery has been extensively studied in other contexts, its role in myelin pathophysiology and repair remains poorly understood. Given the substantial mitochondrial impairment in MS and the significant alterations in intercellular communication mechanisms, including EV-mediated processes, it is plausible that intercellular mitochondrial transfer may also be affected. This hypothesis recognizes neuroinflammatory demyelinating conditions as non-cell-autonomous neurodegenerative diseases involving multiple systems and cellular players.^28^ Moreover, despite promising advancements in mitochondria biotherapy, many questions remain unanswered regarding the molecular pathways governing the complex interplay between mitochondrial dynamics and exchange, neuroinflammation, and their relevance to myelin repair. This review aims to explore our current understanding of the multifaceted mechanisms underlying extracellular mitochondrial transfer in CNS inflammatory conditions associated with metabolic imbalances and its potential implications for myelin repair. Furthermore, we will discuss emerging biotherapeutic strategies—comparing cell- vs cell-free- (naked and EV-encapsulated mitochondria) based strategies for CNS and myelin repair, their challenges and future perspectives for optimization of their clinical applications.

## EV-mediated metabolic regulation in the CNS

EVs are intercellular signaling units that regulate various physiological processes, including neuronal communication, synaptic plasticity, gene expression, immunity, and tissue repair. EVs derived from neural cells carry diverse cargo, including metabolites, enzymes, structural or regulatory proteins, various forms of ribonucleic acid (RNAs), deoxyribonucleic acid (DNA), and intact mitochondria or their components. Mitochondria-containing EVs (Mito-EVs) have been identified in various human body fluids, including blood and cerebrospinal fluid (CSF), under both physiological and pathological conditions.^29^ The intercellular transfer of mitochondrial is of particular interest in energy-deficient contexts, such as demyelination, where restoring metabolic balance is crucial for myelin repair.

In vertebrates, the synaptic release of glutamate following neuronal activity triggers exosome secretion from oligodendrocytes via Ca^2+^ entry through N-methyl-D-aspartate (NMDA) and α-amino-3-hydroxy-5-methyl-4-isoxazolepropionic acid (AMPA) receptors.^30^ Functional internalization of these exosomes cargo by neurons occurs through endocytosis.^30^ EVs released by oligodendrocytes contain specific oligodendroglia proteins, such as sirtuin (SIRT)2, and once internalized by the underlying axon, supports axonal integrity.^31^ SIRT2 deacetylates mitochondrial enzyme ANT (adenine nucleotide translocase) in axons thereby enhancing ATP production^32^ (Figure 1(g)). An in vitro study revealed that increased secretion of small heat shock protein B8 (HSPB8) in human oligodendrocytes EVs resulted in their enhanced internalization by microglia, promoting cellular homeostasis during inflammation revealed by enhancing autophagy markers (LC3B-II, BAG3), reducing oxidative stress, inducing anti-inflammatory effect, mitochondrial depolarization, and ubiquitinated proteins^33^ indicating an immunomodulatory effect of oligodendroglia-EVs on microglia (Figure 1(h)). Such oligodendroglia EV-mediated neurometabolic or immunomodulatory effect represents a potent paracrine mechanism of oligodendrocyte-mediated intercellular communication in the CNS. Weather these communicating oligodendroglia EVs carry mitochondria components remain to be investigated.

Extracellular mitochondria transfer via vesicles (mito-EVs)—including microvesicles (MVs), exosomes, and apoptotic bodies—can mediate the long-distance transport of mitochondria cargo. In vivo studies showed that astrocytes release healthy mitochondria encapsulated by EVs via CD38-mediated mechanism to deliver them to hypoxic neurons and support neuronal mitochondrial metabolism and survival.^34,35^ A recent study reveals that astrocyte-derived EVs transfer mitochondria to brain microvascular endothelial cells and pericytes, a process which increases by ageing.^36^ Hayakawa et al., showed the release of viable mitochondria by endothelial precursor cells and their uptake by brain endothelial cells (BECs), promoting intracellular ATP level, microvascular integrity, angiogenesis and endothelial tightness following an in vitro oxygen-glucose deprivation model of blood-brain barrier (BBB).^21,37^ Interestingly, it has been recently reported that depolarized mitochondria released in mesenchymal stromal cells (MSCs)-derived EVs were internalized by macrophages, where they were restored and recovered their bioenergetic functionality^38^ showing the ability of myeloid cells in recycling mitochondria. Although, the mechanisms behind the packaging of mitochondria into EVs and their uptake or recycling by target cells remain unclear, the oxidative stress and nutrient deprivation signals can trigger these functions.^21^ Regulating CNS metabolic homeostasis can be achieved by either degrading dysfunctional mitochondria released by distressed cells or recycling them and integrating healthy mitochondria into neural cell mitochondrial networks to enhance ATP production and cellular function.^21^

In addition to mito-EVs, mitochondria can produce mitochondrial-derived vesicles (MDVs), which transport mitochondrial components like proteins and lipids. EV markers (such as CD63, Annexin A1 or A2, Alix, TSG101) are not expressed by MDVs.^39^ They are formed from mitochondrial membranes in response to stress signals such as nutrient deprivation, toxins, or oxidative stress.^40^ Interestingly it is reported that MDVs isolated from EVs released by Saccharomyces cerevisiae have a membrane potential, carry selective protein cargo enriched for a functional ATP synthase complex and can produce ATP.^41^ These vesicles can fuse with naive mitochondria, and potentially regenerate ATP-deficient mitochondria via organelle-to-organelle communication. There are two types of MDVs that differ in terms of their structure (single vs bilayer vesicles); origin (outer vs both outer and inner membrane); lipid and protein compositions; or function:^39^ mitochondrial-derived intracellular vesicles (MDIVs), which interact with intracellular organelles like MVBs, peroxisomes, and lysosomes,^42^ and mitochondrial-derived EVs (MDEVs), which are released into the extracellular space. The mechanisms that determine cargo selection are still unclear, however some proteins are known to be selected based on their target destination and the nature of mitochondrial stress.^43,44^ MDIVs destined for lysosomal degradation contain protein kinase PINK1 and cytosolic ubiquitin E3 ligase Parkin, and those following the peroxisome pathway carry the retromer complex and mitochondria-associated protein ligase (MAPL) protein.^45^ The Parkinson’s disease protein parkin tags damaged mitochondrial components for lysosomal clearance.^46^ Additionally, MDEVs can be integrated into EVs transferring materials to extracellular space.^46^ For instance, some proteins such as Optic Atrophy 1 (OPA1) and sorting nexin 9 (Snx9) are required for selective packaging of mitochondria inner membrane or matrix proteins from MDVs and targeting them into EVs.^46^ A recent study revealed that low dose carbon monoxide treatment of oligodendrocytes progenitor cells (OPCs) led to the generation of MDVs that functioned as a preconditioning mechanism to protect the cell from carbon monoxide toxicity^47^ suggesting a protective role for OPC-derived MDVs under pathological condition. Further investigation is needed to understand the roles of MDVs released by different neural cells in CNS and myelin physiology or pathology.

## Targeting mitochondria for myelin repair

As mentioned earlier, CNS has some inherent capacity to compensate the metabolic imbalance in pathological conditions and rescue neurons via transferring healthy mitochondria. Over the past three decades, substantial efforts have been made to halt or reverse MS disease progression. Nevertheless, no cure is currently available, particularly for progressive forms of demyelination. Inspired by key physiological and pathophysiological mechanisms underlying myelin health and disease, several regenerative and innovative biotherapeutic strategies have been investigated and refined, from cell-based therapies to cell-free-based approaches that directly or indirectly target impaired metabolic functions.

### From cell therapy to cell-free approaches in myelin regeneration

Cell-based approaches originally investigated as a strategy to enhance the endogenous myelin repair capacity, replace dysfunctional cells with exogenous myelin-forming cells, or alleviating the inflammatory environment thereby help the insufficient presence of healthy myelin producing cells or providing a permissive environment for the endogenous progenitors to remyelinate the adult CNS.^48,49^ The therapeutic or myelination potential of different cell sources (ranging from primary neural progenitor cells, embryonic stem cells (ES)- or induced pluripotent stem cells (iPS)-derived or directly induced neural or glial progenitor cells, to MSCs or chimeric antigen receptor T (CAR-T) cells recently have been evaluated in different animal models of demyelination with various levels of neuroinflammation (from acute or chronic, focal or systemic, to mutant or humanized mice).^50,51^ The most effective re-myelinating cell sources identified to date have been neural or glial progenitors derived from ES-, iPS cells, or fetal neural/glial progenitors (including patient cells).^49^ These cells have demonstrated the ability to fully re-myelinate developing or adult CNS of myelin-deficient/immune-deficient mouse models following intraparenchymal transplantation.^49^ However, the absence of robust immune-competent animal models replicating key aspects of inflammatory demyelination^52^ has significantly limited advancing these highly re-myelinating xenografts. Moreover, despite significant technical advancements in generating efficient myelin-producing cells, further research is essential to ensure the full safety of these genetically or epigenetically modified cells for human applications.^49^ Furthermore, multiple intraparenchymal injections of potent pro-myelinating cells, which appear necessary for the successful remyelination of the lesions, do not seem practical, particularly in multifocal demyelinating diseases such as MS. Finally, even with successful exogenous remyelination, the highly inflammatory and non-permissive environment of the CNS may pose significant challenges for the long-term survival of transplanted myelin-forming cells, particularly in the context of progressive demyelination, necessitating additional immunosuppressive/immunomodulatory combination therapies or multiple interventions. Thus, despite the promising results achieved concerning the re-myelinating potential of some exogenous cells (including patient own cells) and their beneficial use for preclinical modeling in personalized biomedical research, these complexities vastly limit the feasibility of cell-replacement therapies for adult myelin repair in multifocal neuroinflammatory demyelinating diseases such as MS in clinical settings.

Systemic or intrathecal administration of certain cell sources such as MSCs, allogenic NPCs, or recently CAR-T cells, have shown promising immunomodulatory effects in experimental animal models or in phase I/II clinical trials.^53–56^ The therapeutic effects have been primarily attributed to the ability of transplanted cells to modulate the immune system and enhancing endogenous remyelination via secreting anti-inflammatory or pro-myelinating factors, regulating glial scare formation or neurovascular elements, targeting oxidative stress and mitochondria dysfunction.^57–59^ The exchange of therapeutic cargoes via EVs or mitochondria transfer has been proposed as a potential mechanism of action in some of these cell transplantation studies. EVs released by transplanted MSCs have shown to modulate inflammation, releasing growth factors, stimulating tissue regeneration and alleviating the impaired metabolism in neurodegenerative diseases.^60^ A recent study suggested the EVs and Cx43-gap junction channels as the main mechanisms for mitochondrial transfer from purified MSCs (RECS).^61^ Peruzzotti-Jametti et al., reported that a single intracerebroventricular (ICV) injection of neural stem/progenitor cells (NSCs) results in significant amelioration of EAE clinical scores and disability (as efficiently as NSCs-EVs injection) compared to control mice (received PBS or mitochondria depleted NSCs-derived EVs).^62^ NSCs released respiratory-competent mitochondria (intact or EV-encapsulated) that could integrate into mitochondria network of inflammatory macrophages, modulating their metabolic and inflammatory responses in vitro and in vivo in EAE mice.^62^ Interestingly, postmortem analysis revealed their uptake by different cell types; mainly F4/80+ mononuclear phagocytes (52%) and astrocytes (38%), but also by neurons, oligodendroglia and CD3+ T cells.^62^ Remarkably, brain cells in non-immunized animals were also able to uptake mito-EVs, albeit at a lower percentage compared to EAE animals.^62^ These data suggest that neuroinflammatory demyelinating condition enhances the inherent capacity of astrocytes and mononuclear phagocytes to uptake NSCs-derived mito-EVs in the brain of adult mice.

This evidence has been the primary basis for a shift from cell-based to the cell-free based approaches in some neurological conditions. The strength of EVs/mitochondrial therapies is the perspective to mitigate risks of tumorigenesis, unwanted cell differentiation, inappropriate cell integration, vascular occlusion and shelf-life gains.^63^ Studies show that EVs isolated from adipose-derived stromal cells were able to rescue abnormal expression of mitochondrial proteins in an *in vitro* model of neurodegeneration.^64^ Moreover, transplantation of isolated (free) mitochondria has emerged as a promising cell-free biotherapeutic approach.^65^ The beneficial application of exogenous mitochondria in CNS injuries and diseases has been suggested in several neurological conditions, including stroke, spinal cord injury, schizophrenia, and Parkinson’s disease.^34^ Table 1 summarizes some of the key differences considering cell versus cell-free biotherapies based on EVs/mitochondria transfer. Next sections will further discuss the potential use of cell-free approaches based on EV and mitochondrial biotherapies for myelin repair, the primary challenges in their clinical application, and recent advancements aimed at achieving targeted therapeutic effects.

**Table 1.** Comparing cell vs. cell-free biotherapies based on mitochondria and EVs for myelin diseases.

Feature	Cell-based therapies	Cell-free therapies
Definition	Utilizes live cells (e.g., NPCs, OPCs, MSCs, or CAR-T cells).	Employs subcellular units (e.g., EVs, mitochondria) derived from cells.
Advantages	Replaces damaged cells with functional ones (intraparenchymal injection; NPCs, OPCs). Promotes endogenous remyelination via paracrine signaling (NPCs, OPCs, MSCs). Offers immunomodulatory effects via paracrine signaling (NPCs, OPCs, MSCs, or CAR-T cells)	Lower risk of tumorigenesis or ectopic differentiation. Reduced immune rejection risks. Promotes endogenous remyelination. Offers immunomodulatory effects directly. Simplified logistics, including shelf-life and storage.
Mechanisms of action	Direct remyelination via differentiation into functional myelin (intraparenchymal injection; NPCs, OPCs). Mitochondrial transfer through EVs or direct contact. Immunomodulation (directly or indirectly) by altering inflammatory microenvironments.	Delivery of functional mitochondria (free or EV-encapsulated) to recipient cells. Restoration of bioenergetic and metabolic balance. Immunomodulation via EV cargo, including anti-inflammatory molecules.
Challenges	Risk of tumorigenesis or ectopic differentiation. Limited survival of transplanted cells in highly inflammatory environments. Multiple injections/interventions required for widespread demyelination.	Limited understanding of mechanisms for EV or mitochondrial packaging, release, and uptake, life-time. Potential off-target effects. Standardization of production, preservation, targeted delivery, etc. methods.
Immune modulation	Cells secrete anti-inflammatory factors and modulate glial scarring. May require combination with immunosuppressive therapies to improve survival.	EVs/mito-EVs contain anti-inflammatory molecules and antioxidants. Avoidance of immune rejection with properly prepared cell-free products.
Technical and clinical feasibility	Complex, requiring extensive cell manipulation and immunosuppressive protocols. Ethical concerns with some cell sources (e.g., fetal progenitors).	Easier to produce and standardize. Potential for off-the-shelf therapies. Lower regulatory barriers compared to cell-based methods.
Potential for scale-up	Limited by donor availability and complex cell culture techniques.	High potential for industrial-scale production and long-term storage.

## Mitochondria biotherapy for CNS and myelin regeneration

The transcellular transfer of mitochondria, whether via free, EVs-encapsulated, TNTs or gap junctions, and its specific role in oligodendroglia function and myelin regeneration, remain to be discovered. As mentioned before, the transfer of functional mitochondria is a mechanism used by some exogenous cells to modulate the physiology and metabolism of the target cells.^66^ Transcellular transfer of mitochondria involving different donor and recipient cell types has been recently reviewed elsewhere.^67^ Here, we will review our current understanding of the intercellular mitochondria transfer focusing on free or EV-encapsulated mitochondria in different CNS pathologies including demyelinating or inflammatory conditions.

### EV-encapsulated mitochondria (mito-EVs)

The regenerative potential of mito-EVs released by MSCs have been widely studied recently.^68,69^ EVs carrying mitochondria can be exchanged between neural, neurovascular and immune cells.^70^ Mitochondrial function in the BECs and neurons were improved following exposure to endothelial-derived mito-EVs in vitro and in acute cortical and hippocampal brain slices from mice.^71^ The potential of mito-EVs derived from human BECs have been recently reviewed for their potential in treating ischemic stroke.^72^ In a mouse model of ischemia, intravenously administered medium to large (with mean diameter of 228 nm) EVs containing functional mitochondria isolated from human cerebral microvascular endothelial cell line, significantly reduced brain infarct sizes compared to vehicle-treated mice.^73^ The beneficial effects, safety and underlying mechanisms of mito-EVs therapies in different context and specially in neuroinflammatory demyelination conditions with metabolic imbalance remain to be further investigated.

Blood platelets are often marked as biomarkers in studies on mitochondrial function, neurodegenerative diseases, aging-related conditions, and MS.^74^ Platelet bioenergetics, which reflect mitochondrial function, are increasingly recognized as valuable tools for assessing treatment responses, especially in disorders associated with mitochondrial dysfunction.^75^ Platelets enhance the wound-healing potential of MSCs by transferring mitochondria and inducing metabolic reprogramming, which includes increased *de novo* fatty acid synthesis.^76^ EVs derived from platelets and their mitochondrial content have shown to improve the mitochondrial bioenergetics of cellular immune recipients.^77^ In MS patients, platelets exhibit increased mitochondrial aconitase activity and lipid peroxidation, reduced cytochrome C levels, and elevated expression of mitochondrial superoxide dismutase1 (SOD1), reflecting distinct mitochondrial alterations in MS^78^ (Figure 1(i)). Recent studies have explored the role of aggregated platelets in chronic non-remyelinated lesions in MS and EAE. It has been demonstrated that circulating platelets (found in close proximity to OPCs) enhance differentiation potential of OPCs during remyelination^79^ (Figure 1(a) and (i)). In the EAE model, depletion of circulating platelets impaired OPC differentiation and remyelination but did not affect the numbers or polarization of macrophages and microglia during this process.^79^ Conversely, a sustained increase in circulating platelets was shown to hinder OPC differentiation during remyelination, suggesting a complex, bimodal role of platelets with yet unknown mechanisms^79^ (Figure 1(a) and (i)). Exploring the role of platelet mitochondria or mito-EVs in demyelinated lesions could pave the way for developing targeted therapies to promote myelin repair.

### Free mitochondria delivery

Early studies demonstrated that transplanted mitochondria could transfer antibiotic resistance, such as erythromycin and chloramphenicol resistance, between cells.^80,81^ A new paradigm of mitochondrial therapy based on either local or systemic delivery of mitochondria from autologous or allogenic sources have been stablished.^82^ It aims to restore mitochondrial functions and improve energy metabolism in diseases with metabolic imbalance.^83^ Subsequent research demonstrated that exogenous mitochondria could integrate into recipient cells and enhance their bioenergetic functions.^84^ Various mechanisms have been proposed for the cellular uptake of isolated mitochondria, including caveolae-dependent, clathrin-dependent endocytosis, actin-mediated endocytosis, and micropinocytosis.^85,86^

The therapeutic potential of exogenous free mitochondria has been extensively studied for their ability to regulate energy metabolism and promote regeneration across various models of neurological conditions, particularly ischemic or traumatic injuries, and aging.^84,87,88^ A recent study reported that in vitro transplantation of human platelet mitochondria rescues hypoxia/reoxygenation-induced mitochondrial dysfunction and neuronal cell death involving the FUNDC2/PIP3/Akt/FOXO3a axis.^89^ Nakamura et al. demonstrated the feasibility of using cryopreserved mouse placenta as a source for allogenic mitochondria, with 87% viability, indicating its potential for donor mitochondria transplantation for CNS diseases.^90^ Another study provides evidence on neuroprotective effect of human MSC-derived exogenous mitochondria after ischemia–reperfusion injury by reducing oxidative stress and apoptosis, leading to improvement in motor function and coordination.^88^ Interestingly, in a model of cerebral ischemia, intraparenchymal and unilateral transplantation of exogenous mitochondria purified from allogenic mouse liver, reduced apoptosis, increased OPC proliferation, expression levels of olig2 and MBP and lipid synthesis while improved locomotion recovery 21 days post-injury^91^ showing pro-myelinating effects of liver-derived exogenous mitochondria. Collectively, these findings provide basis for mitochondrial transplantation in treating CNS ischemic injuries^92^ or myelin repair.

Intracerebroventricular transplantation of mitochondria derived from human umbilical cord MSCs improved motor function in a rat model of traumatic brain injury (TBI) through rescuing neuronal cells from apoptosis and alleviating astrogliosis and microglia activation.^93^ Transplantation into the brain lateral ventricle promoted the conversion of microglia from pro-inflammatory M1 to anti-inflammatory M2 phenotypes, improving neuronal survival and cognitive function in mice 24 h after sepsis.^94^ The process also suppressed pro-inflammatory cytokines and boosted endogenous mitochondrial content in M2 microglia. Intravenously (IV) administered human hepatoma cells-derived mitochondria in a model of Parkinson disease, could cross the BBB, were taken up by murine host neuronal cells, boosted ATP production, and promoted cellular proliferation without notable adverse effects.^95^ These studies show the therapeutic effect of ICV or IV administrated exogenous free mitochondria isolated from human (non-neural) cell sources on the rodent CNS cells.

Although extracellular mitochondria transfer has demonstrated therapeutic effects in different CNS conditions, some studies reported deleterious effects or not a significant improvement following mitochondria transfer. Gollihue et al. (2018) reported that local transplantation of mitochondria (isolated from either PC-12 or muscle cells) in a rat model of spinal cord injury, enhanced acute bioenergetics and oxygen consumption rates in a concentration-dependent manner.^84^ However, despite these metabolic improvements, locomotion recovery was not observed. Transplantation of high concentrations of mitochondria resulted in aggregation, triggered inflammatory responses and physical stress at the injection site.^84^ Additionally, they reported that mitochondria can diffuse into white matter more readily than gray matter,^84^ highlighting a differential uptake pattern influenced by tissue structure. Analysis of cell-type-specific localization of GFP+ transplanted mitochondria revealed colocalization with various cell types. The most prominent labeling was observed in endothelial cells and pericytes, with additional localization in brain macrophages, astrocytes, and oligodendrocytes, both at the transplantation site and in distal regions.^84^ However, no evidence of colocalization was detected within neurons.^84^ Therefore, the therapeutic outcomes of extracellular mitochondrial transfer remain complex and context-dependent highlighting the need for further research to optimize mitochondrial delivery methods, concentrations, and targeting strategies to maximize therapeutic efficacy while minimizing adverse effects.

## Clinical applications and the challenges

### State of the art

Advances in some completed and ongoing clinical trials on mitochondrial transplantation, primarily in non-neurological conditions^96^ offer hope for its broader clinical application.^97^ For instance, in an ischemia-reperfusion-induced myocardial dysfunction context, transplantation of autologous mitochondria isolated from the abdominal muscles (via multiple local injection) improved heart function and facilitated withdrawal from extracorporeal membrane oxygenation (ECMO) ststem.^98^ In a recent study focused on safety and efficacy, the same research group demonstrated that intracoronary injection of mitochondria led to rapid uptake and targeted biodistribution of exogenous mitochondria throughout the healthy swine heart.^99^ Several articles have raised key concerns about mitochondrial transplantation, including the viability of mitochondria in the extracellular environment with high Ca^2+^ levels, their ability to effectively and sufficiently produce ATP, or how extracellular ATP can support contraction, and whether naked mitochondria can rapidly cross cell membranes to improve cardiac contractile function.^100,101^ In response to these concerns, McCully et al. (2020) provided evidence supporting the feasibility and effectiveness of mitochondrial transplantation. They noted that (i) cell-free, respiratory-competent mitochondria exist in blood at physiological Ca^2+^ and Na^+^ levels, (ii) multiple studies have shown functional integration of exogenous mitochondria in environments with physiological Ca^2+^ levels, and (iii) transplanted mitochondria increase tissue ATP levels, oxygen uptake, contractile function, and upregulate proteins related to mitochondrial function.^102^ Furthermore, they reported no systemic inflammatory responses following transplantation.^102^

Similarly, mitochondrial transfer enhanced embryo quality and successful births in women with recurrent pregnancy failures,^103^ while limited improvements were observed in children with mitochondrial DNA deletion syndromes.^104^ The safety and efficacy of platelet-derived mitochondrial transplantation via intracoronary injection in 30 patients with acute ST-elevation myocardial infarction (STEMI) have been recently reported in a triple-blind, parallel-group, block-randomized clinical trial.^105^ It is noteworthy that more than 10 clinical trials have recently involved the use of autologous mitochondrial transplantation—primarily using MSC-derived mitochondria—for various conditions, including a clinical trial combining (co-transplanting) MSC-EVs and isolated MSC mitochondria for the treatment of patients with myocardial ischemia (NCT05669144). Altogether, these findings highlight the emerging role of mitochondrial transplantation as a novel therapeutic strategy in regenerative medicine, emphasizing the importance of carefully evaluating and optimizing the mitochondrial source, isolation methods, concentration, and administration protocols.^106^ Additionally, a deeper understanding of the interactions between transplanted mitochondria and various CNS tissues is crucial, including their ability to cross the BBB and their effects on various neural cell types, under different neuropathological conditions. To mitigate potential risks and enhance the therapeutic benefits some of the key challenges for the clinical application of mitochondria transfer are discussed in the following section.

### Challenges and future perspectives in the clinical applications

Biotherapeutic strategies based on mitochondria or mito-EVs, offer promising avenues to mitigate the impact of mitochondria dysfunction in neuroinflammatory demyelination in the CNS. Despite the accumulating body of research showing the beneficial effect of mitochondria (free or EV-encapsulated) biotherapy in vitro or in animal models of different pathological contexts, very few studies examined its effects in models of demyelination and MS. More preclinical studies are needed to explore the therapeutic advantages of extracellular mitochondria in various animal models of MS. On the other hand, existing models may not fully replicate human inflammatory demyelinating conditions or the complexity of the CNS environment.^49^ Moreover, the effectiveness of mitochondrial therapy is expected to be variable among patients due to the heterogeneity of people living with myelin diseases. Finally, the therapeutic success of mitochondrial transplantation relies on factors such as the selection of tissue/cell sources, EV production,^107^ mitochondria isolation and purification protocols, scalability,^108^ quality control, storage, preservation of functional mitochondria, therapeutic dosing, application methods, crossing the BBB, immune-protected transfer, targeted delivery, intracellular destination, toxicity, safety, and ethical and regulatory considerations. Key challenges and future perspectives in applying mitochondrial biotherapy for inflammatory demyelinating conditions are discussed below and summarized in Table 2.

**Table 2.** Challenges and future perspectives in mitochondrial biotherapy for CNS inflammatory demyelination.

Category	Challenges	Future perspectives
Tissue/cell sources	Variability in mitochondrial compatibility, immune responses, and therapeutic potential based on cell source or mitotype. Cell source accessibility.	Identify optimal cell sources for mito-EVs (e.g., NPCs vs OPCs) and assess autologous vs allogenic mitochondria effects.
Immune considerations	Mitochondrial transplantation can have both immunomodulatory and immunogenic effects. High mtDNA heteroplasmy may disrupt cellular homeostasis.	Investigate dose-response effects and immune evasion strategies, including biomimicry and immunosuppressive approaches. Enhance mitochondrial tolerability through mtDNA editing or by coating them with EVs/liposomes.
Mitochondrial isolation and standardization	Current isolation methods are time-intensive and not GMP-compatible. Difficulty in standardizing doses.	Develop rapid, efficient, and scalable GMP-compatible protocols for isolation, characterization, and potency assays.
Storage and preservation	Functional loss during freezing/thawing and storage.	Create methods as off-the-shelf solutions to preserve functionality
Crossing the BBB and delivery	Low uptake across BBB and variability in transport mechanisms. Invasive delivery methods impractical for MS and similar diseases.	Engineer EVs and mitochondria to enhance BBB crossing and develop non-invasive delivery methods (e.g., intranasal).
Encapsulation and coating	Limited uptake and fast clearance. Challenges in carrier production and scalability.	Use engineered and scalable EVs, liposomes, or synthetic materials to enhance mitochondrial delivery and functional stability.
Intracellular fate and integration	Internalized mitochondria risk degradation by lysosomes. Unclear mechanisms for lysosomal bypass.	Utilize fusogenic lipids, EV encapsulation, or artificial lipid membranes to improve cytoplasmic delivery and integration.

### Tissue/cell sources

Despite the general molecular, biogenesis or functional basis of mitochondria or mito-EVs between different cell types, they can be classified based on the cell source or “mitotypes”,^5^ but also based on the cellular differentiation stage, age or condition.^109^ Thus, mitochondria from different sources may vary, which can lead to conflicts with the recipient cell’s endogenous mitochondrial network, affecting cellular metabolism or immune responses. The therapeutic potential of mito-EVs from some of the most used cell sources such as MSCs or platelets (and liver or skeletal muscle cells for isolated mitochondria) have been investigated in different pathological conditions. Some studies indicate that using different sources, such as skeletal muscle, liver, or cell culture, for mitochondrial isolation offers no specific advantage in terms of organ specificity or glycolytic capacity for protection against ischemia-reperfusion injury.^102^ A recent study compared mitochondria transfer between EVs derived from the same species as the recipient cells (e.g., mouse BEC-EVs with mouse BECs) versus cross-species (mouse vs human) EVs and recipient cells.^72^ Results showed that while both hBEC- and mBEC-EVs transferred mitochondria, mBEC-EVs were more effective than hBEC-EVs in enhancing ATP levels and mitochondrial function in recipient mBECs, as well as in reducing brain infarct volume and improving neurological deficits in brain ischemia model in mice.^72^ In the context of neuroinflammatory demyelination, whether extracellular viability, function, compatibility, uptake, integration or adaptation capacity are different for mitochondria derived from various cell source candidates need to be investigated. McCully and colleagues state the autologous tissue obtained from a non-injured site from the patient’s own body offers the most clinically relevant source in cardiovascular coditions.^102^ The potential therapeutic effect of mitochondria from autologous tissues/cells living or struggling in the inflammatory environment of an autoimmune (or genetic) disease must be compared with the allogenic sources. Understanding different types of intercellular mitochondria transfer (contact -dependent or -independent) in the CNS under physiological and pathological conditions is required to identify the most context-based regenerative or deleterious mitotype to target. For instance, although NPCs-derived mito-EVs have the anti-inflammatory and immunomodulatory effects in the EAE model,^110^ whether biomimicry targeting EV-mediated axo-glial interplay by treating the EAE animals with mito-EVs obtained from early stage OPCs or partially differentiated NPCs to OPCs cultures with a presumably closer “mitotypy”^111^ to axo-glial elements can affect the percentage of the mito-EVs uptake by different neural cells or the remyelination and neuroprotection potential are interesting questions to be answered in the future.

### Immune considerations

Mitochondrial transfer therapy offers potential for CNS repair, but its immunogenicity remains a critical consideration.^102^ While some studies report minimal immune rejection following mitochondrial transfer, others highlight the potential for xenogeneic mitochondria—with their bacterial ancestry and unique mtDNA^112^—to trigger immune responses, unlike autologous or allogenic mitochondria.^113^ The impact of mtDNA quality on transplant success is unclear,^114^ as mtDNA can act as a damage-associated molecular pattern (DAMP). Although xenogeneic models are valuable, translational potential is limited by mito-nuclear incompatibility and immune activation.^113^ The routine intravenous administration of platelet transfusions—rich in mitochondria—suggests some tolerance,^115^ though the long-term impact of heteroplasmy requires further investigation.^113^ Mitochondrial transplantation exhibits both immunomodulatory^116^ and immunogenic^114^ effects. Strategies to enhance tolerance could mirror cancer immunotherapy approaches,^117^ modulating immune checkpoints (e.g., PD-1/PD-L1, CTLA-4 pathways). Future research should investigate immune modulation via protein selection, biomimicry, or immunosuppression to improve therapeutic outcomes. Furthermore, techniques such as mitoTALEN-mediated mtDNA editing^118^ and EV or liposome encapsulation could enhance mitochondrial compatibility. Finally, optimizing the mitochondrial cell source, for example, using neuron- or oligodendrocyte progenitor cell-derived mito-EVs, is crucial for maximizing both tolerability and therapeutic efficacy in neural applications.^119^

### Mitochondrial isolation, stability, quality control and standardization

Extracellular mitochondria are highly labile, particularly prone to losing viability or functionality during freeze-thaw cycles. As a result, research on mitochondrial biology and biotherapy has primarily relied on freshly isolated mitochondria. Current methods for mitochondrial isolation and characterization are often time-intensive, potentially compromising mitochondrial quality and reducing the likelihood of successful transplantation.

The most used mitochondria isolation techniques are based on differential centrifugation or differential filtration. It has been reported that isolated mitochondria using a rapid protocol (30 min isolation) via differential filtration remain stable at 4°C for up to 120 minutes with no significant changes in mitochondrial oxygen consumption rate up to 2 h post-isolation.^102^ However, a significant decrease in oxygen consumption rate was detected at 180 minutes. Other techniques include magnetic-based isolation and mitochondria isolation kits. Most of these techniques are not GMP-compatible or suitable for the large-scale production, thus have limitation for the clinical applications.

Moreover, the absence of a simple, rapid, reliable, and universally accepted quantification and characterization method or potency assays, has hindered the standardization of mitochondrial doses for transplantation.

During isolation, storage, and delivery, mitochondria can lose membrane potential or undergo oxidative damage, reducing their efficacy. Frozen mitochondria with reduced oxygen consumption and membrane potential fail to protect against ischemia-reperfusion injury.^65^ Additionally, thawed, disrupted or mitochondria membrane particles or organelle secretions were shown to be ineffective in various pathological context.^120,121^ Several techniques have been developed to improve the preservation of mitochondria for later use. These techniques focus mainly on reducing damage during the freezing process or maintaining mitochondrial integrity and function after thawing. Some methods include using cryoprotectants (such as dimethyl sulfoxide (DMSO), terhalose, glycerol), controlled rate freezing, membrane coating, optimization of thawing protocols and encapsulation in EVs.^122^ Ideally, an “off-the-shelf” product without functional disruption for immediate use or clinical application is desired, but despite some efforts for their preservation,^123,124^ the methodology for manufacturing such a product has not yet developed. Consequently, the use of freshly isolated mitochondria has been the only option for free mitochondrial biotherapy.

### Encapsulated and coated mitochondria transfer and delivery

The biotherapeutic potential of EVs capable of carrying and delivering functional mitochondria in EAE models has provided new insights into mitochondrial-based therapies.^62^ EVs are remarkably stable in the extracellular environment due to their protective lipid bilayer membrane, which shields their cargo from extracellular enzymatic degradation, immune clearance, oxidative or freezing-thawing stress, and environmental challenges such as variations in pH, osmolarity, and electrolytes. Mitochondria encapsulated within EVs exhibit greater resistance to Ca^2+^ overload and oxidative stress compared to isolated mitochondria.^125^ EVs can protect against immune activation associated with extracellular mitochondria and their components as DAMPs,^126,127^ and help deliver cargo over long distances.^111^ However, despite these advantages, several challenges remain including low-yield EV production, purification, scalability, EV heterogeneity, biodegradation and therapeutic dosages. A recent study revealed that ∼30% of MSC-released EVs contained mitochondria.^128^ Other studies showed that only medium to large size (containing functional mitochondria) but not small MSC-EV fractions could have therapeutic effect.^73^ Moreover, EVs can be engineered to optimize their cargo loading,^129^ target-specific delivery, endosomal escape mechanisms, and therapeutic efficacy.^130^ Treatment of NIH/3T3, a fibroblast cell line and hCMEC/D3, a human BEC line with PGC-1α (resveratrol, activated peroxisome proliferator-activated receptor-gamma coactivator-1α), which is a key mediator of mitochondrial biogenesis, increased release of mito-EVs, enhanced ATP levels and transferred mitochondria to recipient BECs.^131^ This highlights how pharmacologically modulating donor cell mitochondrial biogenesis can enhance mitochondrial content in secreted microvesicles. However, despite these advancements, significant challenges still remain unanswered.

Different mechanisms have been described by which mitochondria can enter the cells with endocytosis being a common way.^132^ Unmodified EV and mitochondria therapeutics usually suffer from their limited uptake and fast clearance in target tissues. Different approaches have been proposed for improving mitochondria encapsulation^127,133^ or delivery purposes. These include using membrane-penetrating peptides,^119^ dextran triphenylphosphonium-based polymer,^133^ droplet-based microfluidics,^134^ liposomes^135^ either to enhance mitochondrial load of cells in vitro,^136^ or to improve their in vivo therapeutic effects.^137^ A review article comparing liposomes and EVs highlights similarities in cellular uptake via endocytosis and intracellular accumulation,^138^ though a side-by-side comparative research on their delivery efficacy and therapeutic potential remains elusive. A recent study shows that encapsulation of mitochondria by Zeolitic Imidazolate Framework-8 (ZiF-8), a type of Metal–Organic Framework (MOF) material used for coating cells or viruses, has improved the metabolic function of M2 type macrophages.^139^ Additionally, simple modification of mitochondria with a sugar such as O-GlcNAcylation has been suggested to protect against mitochondria antiglycation, autophagy and oxidative stress stimulation via RAGE (receptors for advanced glycation end product).^140^ Moreover, whether lyophilized EVs^141^ or vitrification methods could keep the viability and functionality of their mitochondria cargo need to be investigated. Therefore, engineering and coating of isolated mitochondria by synthetic or biogenic materials such as liposomes or EVs^142^ may help keeping their functionality while minimizing the possible adverse effects of mitochondria transplantation.

### Crossing the BBB and mode of delivery

Different application modes (such as intranasal, intracerebroventricular, intrathecal or intravenous, deliveries), may differently affect the rate of BBB uptake of the EVs.^143^ Invasive or locally administrated approaches may not be practical for multifocal inflammatory demyelinating diseases, especially if multiple injections are needed periodically. Under physiological condition, systemically administered materials struggle to cross the BBB, with low brain uptake and rapid clearance. Several conditions including inflammatory demyelination and ischemic stroke compromise the BEC function and BBB integrity. Targeting BBB integrity has been a treatment strategy for CNS inflammatory diseases such as MS.^144^ However, modeling the BBB for in vivo studies under physiological or pathological conditions remains challenging. In vitro studies also lack a dynamic BBB, limiting their applicability in designing mito-EV-based strategies for CNS biotherapies. Although mito-EVs can cross the BBB under pathological condition,^145^ but their crossing rates and transport mechanisms may vary, affecting treatment efficacy.^146^ Therefore, engineering methods have been employed to allow the modification of EVs or mitochondria to prolong their circulation in the blood, enhance brain uptake and their signaling action. Exogenous BEC-derived mito-EVs support BEC cell survival integrity through mitochondrial transfer.^71^ A recent study used a mixtures of EV/exogenous heat shock protein (HSP27) strategy to increase BEC survival (via mito-EVs) and preserve their tight junction integrity (via HSP27 effects).^73^ Formulations of EVs with HSP27, with or without the addition of a cationic polymer (PEG-DET), effectively reduced paracellular permeability in oxygen-glucose-deprived BECs.^73^ Design of several engineered EVs methods to increase EV uptake by BBB have been reviewed recently.^147^ A recent study developed a mitochondrial compound targeting macrophages at spinal cord injury (SCI) sites. Mitochondria were isolated from IL-10-induced Mertk^hi^ macrophages and were conjugated with a peptide sequence, cations-cysteine-alanine-glutamine-lysine (CAQK), that can effectively target proteoglycan compounds in the injured center. The compound enhanced macrophage phagocytosis of myelin debries, reduced lipid buildup, restored mitochondrial function, and suppressed inflammation in vitro and in vivo.^148^ Intravenous delivery effectively targeted the SCI epicenter, with macrophages being the primary recipients, promoting tissue regeneration and functional recovery in mice.^148^

### Intracellular fate and integration

Internalized mitochondria (free or encapsulated) need to be functionally incorporated into mitochondria network of the recipient cells, such as oligodendrocytes, astrocytes, neurons or microglia for bioenergetic and repair benefits. Several mechanisms have been described for mitochondria or EVs uptake by the cells. It remains unclear whether mitochondria need to be internalized by cells to improve disease conditions or if they can exert therapeutic effects as extracellular mitochondrial vesicles/particles.^149^ Internalized mitochondria are often recognized as foreign and may be degraded by lysosomes.^4^ Functionalizing isolated mitochondria with a biocompatible polymer (dextran-triphenylphosphonium) enhanced mitochondrial protection, cellular internalization, and improved oxidative phosphorylation in cancer and cardiovascular cells.^133^ The mechanisms by which some signals can bypass this lysosomal degradation is not totally understood. Efforts have been made to protect mitochondria from intracellular lysosomal degradation.^150^ A recent study showed that lysosomal inhibition triggers the secretion of intact mitochondria enclosed within large EVs.^151^ Deletion of the small GTPase Rab7 in cells or the adult mouse heart increased the secretion of EVs containing ubiquitinated cargos, including intact mitochondria.^151^ The released EVs were taken up by macrophages without inducing inflammation.^151^ Another study showed that mitochondria transfer using fusogenic lipids can bypass lysosomal degradation.^152^ Fusogenic lipids facilitate direct mitochondrial transfer into the cytoplasm by fusing with the cell membrane to form a channel.^152^ Additionally, coating mitochondria with an artificial lipid membrane 1,2-dioleoyl-3-trimethylammoniumpropane/dioleoylphosphatidylethanolamine (DOTAP/DOPE) has been shown to enhance cellular uptake, stabilize mitochondrial membrane potential, preserve mitochondrial function, and improve neuroprotection in cultured neurons and a mouse model of cerebral ischemia-reperfusion.^127^ However, mitochondria coating or modification after isolation is time-consuming and may destabilize the lipid bilayer of mitochondria^152^ and provoke harmful effect. It has been reported that N-acetylcysteine amide (NACA), a mitochondrial glutathione regulator, helps preserving mitochondrial bioenergetics in spinal cord injury^153^ or prevents mitochondria impairment in heart and kidney when administrated before the injury.^154^ A recent study identified endosomal escape proteins (similar to that in viruses^155^) in EVs allowing them, under specific conditions to safely deliver their cargo within the host cell.^156^ Thus, encapsulating mitochondria by engineered EVs or fusogenic liposomes may help them to bypass lysosomal degradation.

## Conclusions

CNS demyelination is a hallmark of various neuroinflammatory and degenerative conditions, including MS, where mitochondrial dysfunction in oligodendroglia, neurons, astroglia, microglia, or immune cells is associated with intercellular molecular disruption, metabolic decoupling, and bioenergetic imbalance. Whether intercellular miscommunication or mitochondria dysfunction is a cause or a consequence of neuroinflammatory demyelination is not known. Despite significant advancements in disease-modifying therapies, mainly through immunomodulatory compounds, there are currently no treatments available to halt or reverse progressive demyelination. Myelin-forming precursor cells are present in MS lesions, but their differentiation into mature myelinating oligodendrocytes is impeded for reasons that remain poorly understood. Widespread mitochondrial dysfunction and metabolic imbalance have driven efforts to target the endogenous precursor pool, aiming to enhance their metabolic support, alleviate the inflammatory microenvironment, promote remyelination, and prevent axonal loss. In parallel, several cell-based biotherapeutic approaches, such as allogenic NPCs/NSCs, OPCs or MSCs, have emerged to help manage the lost axoglial functions. These approaches have shown promise in modulating the immune system or replacing lost myelin in various animal models. However, challenges such as safety concerns and delivery methods have largely limited their clinical application, particularly for the myelin replacement therapies. Furthermore, the lack of a comprehensive animal model has made it difficult to fully investigate the immunomodulatory and remyelinating potential for some of these cell sources.

The discovery of mito-EVs released by transplanted NPCs in a chronic EAE model has shed light on the mechanisms underlying the observed immunomodulatory effects of transplanted cells.^62^ Building on advances in understanding EV- and mitochondria-mediated intercellular metabolic communication, which supports neural homeostasis under physiological conditions, research is increasingly focusing on targeting these signaling pathways. The therapeutic potential of mitochondrial biotherapy in both neurological and non-neurological conditions has propelled its progression into numerous clinical trials worldwide. However, the clinical application of mitochondrial therapy for neuroinflammatory neurological conditions is still in its early stages, requiring further context-dependent optimization and validation to maximize therapeutic efficacy while minimizing risks. Key considerations include selecting good manufacturing practice (GMP)-compatible cell sources, optimizing isolation and storage methods, preserving functional integrity, protecting against immune responses, identifying the best encapsulation or coating strategies, achieving large-scale carrier production, ensuring targeted delivery and cellular uptake, preventing endosomal degradation, conducting potency assays, enabling BBB crossing, and ensuring functional integration into recipient CNS cells. Successful implementation of these steps should help restore metabolic homeostasis, promote immunomodulation, reestablish intercellular communication networks and improve endogenous remyelination.

Encapsulated mitochondria within biogenic or synthetic carriers, such as EVs or liposomes, hold great promise for treating inflammatory demyelinating diseases characterized by mitochondrial dysfunction and intercellular metabolic decoupling. However, further research is needed to fully elucidate intercellular mitochondrial communication and the complexity of intercellular-mediated metabolic imbalances in neuroinflammatory demyelinating diseases. Finally, given the critical role of mitochondria in the CNS physiology and pathology, it is essential to determine whether transcellular mitochondrial transfer promotes health or contributes to disease. Addressing these challenges will require interdisciplinary collaboration across neuroscience, biomedical engineering, and translational/clinical research to optimize encapsulated mitochondrial therapies for effective myelin repair at clinics.
